# Consumption and Breakfast Patterns in Children and Adolescents with Congenital Heart Disease

**DOI:** 10.3390/ijerph20065146

**Published:** 2023-03-15

**Authors:** Joanna Maraschim, Michele Honicky, Yara Maria Franco Moreno, Patricia de Fragas Hinnig, Silvia Meyer Cardoso, Isabela de Carlos Back, Francilene Gracieli Kunradi Vieira

**Affiliations:** 1 Post-Graduation Program in Nutrition, Federal University of Santa Catarina, Florianópolis 88040-900, Brazil; 2 University Hospital Polydoro Ernanni de São Tiago, Federal University of Santa Catarina, Florianópolis 88040-900, Brazil; 3 Postgraduate Program in Public Health, Health Sciences Center, Federal University of Santa Catarina, Florianópolis 88040-900, Brazil

**Keywords:** breakfast patterns, skipping breakfast, cardiovascular diseases, pediatrics, congenital heart disease

## Abstract

Little is known about skipping breakfast and breakfast patterns (BP) and their evaluation according to sociodemographic, clinical, lifestyle, cardiometabolic and nutritional data in children and adolescents with congenital heart disease (CHD). This cross-sectional study with 232 children and adolescents with CHD identified the prevalence and patterns of the breakfast, described these according to sociodemographic, clinical and lifestyle characteristics, and assessed their association with cardiometabolic and nutritional markers. Breakfast patterns were identified by principal components, and bivariate and linear regression analysis were applied. Breakfast consumption was observed in 73% of participants. Four BP were identified: pattern 1 “milk, ultra-processed bread, and chocolate milk”, pattern 2 “margarine and processed bread”, pattern 3 “cold meats/sausages, cheeses and butter/cream” and pattern 4 “fruits/fruit juices, breakfast cereals, yogurts, and homemade cakes/pies and sweet snacks”. Family history for obesity and acyanotic CHD were associated with breakfast skipping. Younger participants and greater maternal education were associated with greater adherence to pattern 1 and pattern 4. Older participants and longer post-operative time showed greater adherence to pattern 3. No association between skipping breakfast or BP and cardiometabolic and nutritional markers was observed. Nonetheless, the findings reinforce the need for nutritional guidance for healthy breakfast, aiming to reduce the consumption of ultra-processed foods and to prioritize fresh and minimally processed foods.

## 1. Introduction

Children and adolescents with congenital heart disease (CHD) are more likely to develop overweight or obesity and changes in cardiometabolic markers, such as increased blood pressure [1]. This is possibly due to a sedentary lifestyle [2,3] and high caloric and ultra-processed foods consumption [4,5,6].

Feeding behavior during childhood plays an important role in preventing excess body weight and changes in metabolic markers related to developing acquired cardiovascular diseases in adulthood [7]. Evidence indicates that breakfast is often recognized as the most important meal of the day, even though there is no consensus in the literature about its definition and ideal composition of nutrients and foods [8,9]. Furthermore, studies describe the association between skipping breakfast and obesity [10,11] and changes in cardiometabolic markers or metabolic syndrome in children and adolescents [8,11,12,13].

Besides having breakfast, the composition of this meal has important implications for cardiometabolic health [14], considering that foods are consumed together and not in isolation [15]. Thus, different methods for assessing breakfast quality have been defined [8,16]. The identification of dietary patterns (DPs) emerged as an assessment alternative, making it possible to know the DPs and their associations with a particular disease [17]. The association between breakfast patterns with obesity and cardiometabolic risk factors has already been well studied in adults [18,19,20]. However, few studies have been carried out with healthy children and adolescents, which identified breakfast patterns and evaluated their association with overweight and obesity [16,21].

In children and adolescents with CHD, as far as we know, there has been only one study carried out in Brazil, which identified six DPs of the global diet and observed that an unhealthy DP (poultry, red meat, cold cuts, and processed meats, soft drinks and sweetened beverages) was associated with a higher risk of central adiposity, a healthy DP (fish, eggs, bread, beans, tubers and roots, fruits and fruit juice) was associated with a decreased risk of central adiposity, and a low dairy DP (milk and dairy products with low-fat content, mixed dishes, ultra-processed bread, sweets, and chocolate) was inversely associated with carotid intima-media thickness [22].

In this context, the primary objectives of this study were to identify the prevalence of skipping and breakfast consumption, to describe the breakfast patterns, and to evaluate the sociodemographic, clinical and lifestyle characteristics according to the skipping and breakfast consumption and breakfast patterns of children and adolescents with CHD. Second, we aimed to investigate the association of skipping breakfast and breakfast patterns with cardiometabolic and nutritional markers in this population.

## 2. Materials and Methods

### 2.1. Study Design and Population

A cross-sectional study was carried out with children and adolescents with CHD previously undergoing a cardiac procedure, who were monitored in pediatric cardiology outpatient clinics in two reference hospitals in southern Brazil, from January to July 2017. 

Sample size calculation was performed using the OpenEpi^®^ 3.01 software (Atlanta, GA, USA) based on the study outcome variables (cardiometabolic markers and nutritional status), assuming a type 1 error (α) of 0.05, type 2 error (β) of 0.20, and 95% confidence interval. Considering that the highest prevalence among outcomes was 26.9% obesity, as assessed by air displacement plethysmography (Bod Pod^®^ Body Composition System, COSMED, Concord, CA, USA) in Brazilian children and adolescents with CHD [23], the study required a sample with 131 children and adolescents with CHD. 

Inclusion criteria were: (I) age between 5 and 18 years; (II) diagnosis of CHD; and (III) after therapeutic catheterization or cardiac surgery for CHD. Exclusion criteria were: (I) secondary diagnosis of malignant neoplasm; (II) chromosomal anomalies; (III) primary or secondary familial dyslipidemia; (IV) diabetes mellitus or hypothyroidism; and (V) presence of acute or chronic inflammatory diseases in the last 15 days. 

The data are from the Floripa CHild study (Congenital Heart dIsease and atheroscLerosis in chilDren and adolescents Study), a longitudinal study aiming to investigate risk factors for atherosclerosis in children and adolescents with CHD.

This research was approved by the Research Ethics Committee at Joanna Gusmão Children’s Hospital, Brazil (no. 1.672.255/2016) and was conducted following the Declaration of Helsinki. The children and adolescents obtained written authorization from their legal guardians and agreed to participate in the study. 

### 2.2. Dietary Assessment

The assessment of food consumption was based on three 24 h recalls on non-consecutive days (two on weekdays and one on weekend), using the multiple-pass technique [24]. The first 24 h recall was applied at the time of data collection, and the next two were obtained via telephone, with an interval between recalls of 7.3 weeks (SD 3.21). Details on the collection and processing of recalls are available in previous studies [22,23]. 

A photographic album to aid in reporting the portion sizes of food intake was used [25]. The recalls were entered into the Nutrition Data System for Research^®^ (NDSR) software, 2017 version (University of Minnesota, Minneapolis, MN, USA), which includes the name and meal times to obtain specific food consumption data by meals. This software uses the United States Department of Agriculture (USDA) database as the main database. Initially, the nutritional equivalences of the foods available in the software were checked on Brazilian charts [26], and Brazilian typical recipes were entered manually into the software [27]. The data of the foods and preparations (g or mL) from the 24 h recall were entered into the NDSR software after using standardized methodology and Brazilian home measurement charts [28,29].

### 2.3. Breakfast Consumption Definition 

This study adopted the concept of breakfast based on guardians of children and adolescents identification during the application of 24 h recalls [30]. Breakfast skipping was considered as not having breakfast on at least one of the three days evaluated based on the definition of skipping this meal at least once per week as used by others [31,32].

### 2.4. Identification of Breakfast Patterns

The breakfast patterns were generated only with data from participants who had this meal in the three 24 h recalls. One hundred twenty-seven breakfast food items were reported. Foods with consumption frequency lower than 5% were excluded from the analysis.

Food items were grouped based on the similarity of nutritional composition and their respective degrees of processing, according to the NOVA [33] classification (UP, ultra-processed; P, processed), resulting in 19 food groups (Table 1). Data in grams were adjusted for the total energy intake using the residual method [34].

The breakfast patterns were derived by principal component analysis. The Kaiser–Meyer–Olkin (KMO) statistical test was performed to verify the applicability of the factor analysis, and a KMO value of 0.55 was considered acceptable [35]. The eigenvalues of 1.30, the screeplot [35,36] graphical representation, and the interpretation of breakfast patterns by nutritionists were considered to retain the number of factors. Varimax rotation was used to simplify data interpretation, and the food groups with factor loadings |≥0.25| were considered representative of each breakfast pattern [36].

For each component retained, a score was generated for each participant. The score was calculated considering the amount in grams of each food group multiplied by the factor loading of this item in the pattern, with higher scores corresponding to greater adherence to a specific breakfast pattern.

### 2.5. Sociodemographic, Clinical and Lifestyle Data

Sociodemographic data such as age (in years), sex (female/male), mother’s education (<10 years of schooling and ≥10 years of schooling), and family history for obesity (absent/present) were collected.

Clinical information such as the classification of congenital heart disease (cyanotic/acyanotic) [37] and the mean post-operative time (in years) were collected from the medical records.

Sedentary behavior was assessed by the number of hours spent leisurely in front of the television, computer/similar and/or electronic games and time spent sitting per day, categorized as no, <8 h/day, and yes, ≥8 h/day, and in hours/day [38].

### 2.6. Nutritional Markers

Waist circumference (cm) was measured with the participant in the standing position on the iliac crest and at the end of a normal expiration [39], using a non-elastic tape with 0.1 cm precision (TBW^®^, São Paulo, Brazil), by a trained nutritionist. The waist circumference percentiles for sex and age were calculated [40].

Body composition was performed using air displacement plethysmography (Bod Pod^®^ Body Composition System, COSMED, Concord, CA, USA) following the calibration procedures described by the manufacturer [41]. Details of the test protocol have been described previously [23]. The software determined the percentage of body fat (%) and the percentage of lean mass (%) using the calculation proposed by Lohman (1989) [42] for children and adolescents.

### 2.7. Cardiometabolic Markers

Fasting glucose concentration was assessed using the colorimetric enzymatic method and was expressed in mg/dL. Total cholesterol and triglycerides were determined by the enzymatic method (Dimension^®^, Siemens, Newark, NJ, USA). The HDL-c concentration was obtained by the direct method, in vitro. Non-HDL-c values were obtained by subtracting total cholesterol from HDL-c. LDL-c concentrations were calculated using the Friedewald formula [43]. Lipid parameter values were expressed in mg/dL. The plasma concentration of hs-CRP was determined by immunonephelometry (BN II^®^, Siemens Healthcare Diagnostics Inc., Newark, DL, USA), expressed in mg/L. The measurement of systolic and diastolic blood pressure was performed with a mercury sphygmomanometer and an appropriate cuff according to the arm circumference (Tycos, Welch Allyn^®^ New York, NY, USA), following the protocol of the National High Blood Pressure Education Program Working Group on High Blood Pressure in Children and Adolescents (NHBPEP), expressed in percentiles and calculated according to sex, age, and height percentile [44].

### 2.8. Statistical Analysis

The Kolmogorov–Smirnov test, coefficient of variation, and histograms were performed to assess the normality of the data. Descriptive data were presented as mean and standard deviation (SD) or median and interquartile range (IQR) and relative and absolute frequencies.

To assess the sociodemographic, clinical and lifestyle sample characteristics according to adherence to the breakfast pattern, the scores of each one of the four breakfast patterns were categorized as below or above the median, with children and adolescents with CHD above the median showing greater adherence to the breakfast pattern. In the bivariate analysis, between the breakfast consumption or the breakfast patterns and sociodemographic, clinical and lifestyle sample characteristics, the chi-square test and Student’s *t* test were used. 

Simple and multiple linear regression by the forward selection procedure was performed to investigate the association between breakfast skipping or factorial scores of each breakfast pattern (independent variables) and cardiometabolic and nutritional markers (dependent variables). Asymmetric variables were transformed into logarithmic and later into exponential numbers. The covariates considered in multiple analyses were based on the bivariate analysis (*p* < 0.20) and potential confounders for cardiometabolic and nutritional markers described in the literature [37,45]. The variance inflation factor (VIF) was used to analyze the collinearity between the variables, (VIF) > 10 were not included, avoiding multicollinearity. Multivariable-adjusted analysis was adjusted for age (in years), sex (female/male), maternal education (<10 years and ≥10 years) (Adjustment 1), adjustment 1 + sedentary behavior (in hours/day), postoperative period (in years), family history for obesity (absent/present), classification of congenital heart disease (cyanotic and acyanotic and according to the international code of diseases (ICD-10)), waist circumference (percentile) (Adjustment 2), and adjustment 2 + glucose (mg/dL) (Adjustment 3), only for HDL-c and non-HDL-c variables. Linear regression models between breakfast skipping and cardiometabolic and nutritional markers were also adjusted for total daily energy (kcal/day) [34]. Total daily energy was adjusted for intra-interpersonal variability [46]. The results were expressed in regression coefficients and respective 95% confidence intervals (95% CI). All statistical analyses were performed using Stata^®^ software version 13.0 (STATA Corporation, College Station, TX, USA).

## 3. Results

### 3.1. Characteristics of Study Participants

Three hundred nineteen children and adolescents were considered eligible for the study, of which the following were excluded: not contacted (n = 63), chromosomal syndrome (n = 7), under five years old (n = 4), over 18 years old (n = 12), and losses (nephrotic syndrome n = 1). Thus, 232 children and adolescents with CHD participated in the study. Supplemental Figure S1 shows the flowchart of participant selection. The mean age was 10.2 (SD: 3.7 years), 52.5% were girls, 63.4% had sedentary behavior, the mean postoperative time was 6.7 (SD: 3.8 years), 35.5% of the participants had a family history for obesity, and 66% with acyanotic CHD.

Of the 232 children and adolescents with CHD, 73% had breakfast consumption. The mean energy intake for breakfast among children and adolescents with this meal was 78.3 (SD: 53.8 kcal).

Among participants who skipped breakfast (27%), most were girls (57.1%), had a family history for obesity (52.4%), and 66.6% had acyanotic CHD. Bivariate analysis showed that participants with a family history for obesity (*p* = 0.001) and those with acyanotic CHD (*p* = 0.01) were associated with breakfast skipping. Characteristics of the total study population according to skipping and breakfast consumption are shown in Table 2.

### 3.2. Breakfast Dietary Patterns

Four breakfast patterns were identified, which explained 37.0% of the total breakfast variability. Breakfast pattern 1 was characterized by high consumption of milk, UP bread, and chocolate milk, and low consumption of homemade cakes/pies and sweet snacks and coffee/tea. Breakfast pattern 2 included high consumption of margarine and P bread. Breakfast pattern 3 included high consumption of cold meats/sausages, cheeses, butter/cream, and low consumption of sugary drinks and soft drinks. Breakfast pattern 4 was characterized by high consumption of fruits/fruit juices, breakfast cereals, yogurts, and homemade cakes/pies and sweet snacks, and low sugar, coffee, and tea (Table 3).

In the bivariate analysis, there was an association of younger participants with greater adherence to the breakfast pattern 1 of milk, UP bread, and chocolate milk (*p* = 0.001) and to the breakfast pattern 4 of fruits/fruit juice, breakfast cereals, yogurts, and homemade cakes/pies and sweet snacks (*p* = 0.001), and older participants showed greater adherence to the breakfast pattern 3 of cold meats/sausage, cheeses and butter/cream (*p* = 0.003). Maternal education was associated with the breakfast pattern 1 of milk, UP bread, and chocolate milk (*p* = 0.01) and the breakfast pattern 4 of fruits/fruit juices, breakfast cereals, yogurts, and homemade cakes/pies and sweet snacks (*p* = 0.002), with participants whose maternal education level was greater than 10 years showing greater adherence to these patterns compared to participants with lower maternal education. In addition, the longer post-operative time of the participants was associated with greater adherence to the breakfast pattern 3 of cold meats/sausage, cheese, and butter/cream (*p* = 0.04) (Table 4).

### 3.3. Association Analysis between Skipping Breakfast and Breakfast Patterns with Cardiometabolic and Nutritional Markers

In the multiple linear regression analysis, after adjustments for confounding factors (Adjustments 1 and 2), no association was found between skipping breakfast and cardiometabolic markers and nutritional status markers (Table 5).

The cardiometabolic and nutritional markers were also not associated with any of the four breakfast patterns in the multivariate analysis adjusted for potential confounding factors (Adjustments 1, 2, and 3) (Table 6).

## 4. Discussion

To date, this is the first study that identified skipping, breakfast consumption and the breakfast patterns and assessed their association with sociodemographic, clinical and lifestyle characteristics as well as cardiometabolic and nutritional markers in children and adolescents with CHD. In this cross-sectional study carried out in southern Brazil with children and adolescents with CHD, 27% of the participants skipped breakfast. Breakfast skipping was associated with a family history for obesity and participants with acyanotic CHD. Among those who consumed breakfast, four breakfast patterns were identified. The first pattern characterized by the high consumption of milk, UP bread, and chocolate milk showed greater adherence in younger patients and between those with higher maternal education. The second pattern, constituted by the high consumption of margarine and processed bread, was not associated with any analyzed variables. The third pattern characterized by the high consumption of cold meats/sausages, cheeses, and butter/cream showed greater adherence among older patients and with a longer mean postoperative time. The fourth pattern characterized by the high consumption of fruits/fruit juices, breakfast cereals, yogurts, and homemade cakes/pies and sweet snacks showed greater adherence in younger patients and those with higher maternal education.

The frequency of breakfast consumption among the participants in this study was 73%, similar to that found in previous studies carried out in Brazil (79 to 91%) [47,48,49], Jordan (80%) [50] and Mexico (83%) [15] but higher than those identified in the United States (14–18.7%) [16,51] and in Spain (5.3%) [8]. In this study, 27% of the participants skipped breakfast, lower than that found between adolescents in other Brazilian studies (36.2% to 38%) [52,53]. Furthermore, we found a higher prevalence of breakfast skipping among participants with a family history for obesity (52.4%), similar to a study in Egypt [54], and despite that a higher prevalence of breakfast skipping among participants with acyanotic CHD (66.6%) was observed, no similar study was found for comparison.

In this study, no association was found between breakfast consumption and cardiometabolic and nutritional markers. This fact can be partially explained by our definition of breakfast, as we did not consider the occasional consumption of breakfast. In a study in Southern California [55] of overweight adolescents with a family history for type 2 diabetes, it was observed that breakfast consumption was associated with increased intra-abdominal adipose tissue. Breakfast was defined as any food or drink consumed between 5:00 and 10:00 a.m. with a total combined energy ≥100 kcal, and people who consumed breakfast occasionally were also included [55]. In another study in Taiwan [13] with elementary school students, breakfast was defined with the question “how often do you eat breakfast in the week?”. It was found that children who consumed breakfast daily had lower risks of high blood pressure and metabolic syndrome compared to children who consumed breakfast 0 to 4 times per week [13]. Similar to our results, a study in the Netherlands [56] did not observe an association between breakfast skipping and being overweight in children aged 2 to 5 years. The authors did not consider occasional breakfast consumption. 

The breakfast pattern 1 “milk, UP bread and chocolate milk” identified in this study confirms the results of the research by Marchioni et al. (2015) [53], demonstrating that milk, UP bread, and chocolate milk are among the most consumed foods for breakfast among healthy Brazilian adolescents. Similar results were shown in international studies, as observed in healthy Mexican children, in which the milk and sweetened bread pattern was the most consumed foods for breakfast [15]. Studies with healthy children in Greece [57] and Spain [58] found that milk and chocolate milk were the most frequently consumed foods for breakfast. In addition, greater adherence to the pattern “milk, UP bread and chocolate milk” by younger participants was observed in this study, in line with a previous study carried out in Brazil [59], which identified a higher prevalence of consumption of foods such as milk at breakfast (63.3%), bread (59.5%), dairy products (3.3%), and chocolate milk (29.1%) among children aged 7 to 9 years old.

The breakfast pattern most consumed by children aged 9 to 11 years in France [60] included mainly flavored milk, bread, fat (butter), and juice, similar to the breakfast pattern 2 of “margarine and P bread” found in this study. The food groups found in this pattern are similar to those identified in the breakfast of Brazilian adolescents [61].

The breakfast pattern 3 of “cold meats/sausages, cheeses and butter/cream” in this study corroborates what was observed in a study carried out with schoolchildren aged 7 to 13 years in southern Brazil, in which the “Traditional Brazilian Pattern” was made up of bread, cheese, sausages, and coffee with milk, which was the most consumed among the three identified breakfast patterns [62]. In addition, a similar result was found in a population-based study carried out with adolescents aged 10 to 19 years in Brazil, in which the breakfast pattern was protein based, consisting of cold cut meat, milk and cheese [21]. In addition, greater adherence to the pattern “cold meats/sausages, cheeses and butter/cream” was identified in participants with a longer mean postoperative time. However, no similar study was found for comparison. 

A similar result to breakfast pattern 4, “fruits/fruit juices, breakfast cereals, yogurts, and homemade cakes/pies and sweet snacks”, was found in a study carried out in the United States [16] with healthy children and adolescents, which identified the breakfast pattern of ready-to-eat cereals and whole milk. In Mexico [15], of the six breakfast patterns identified in children aged 4 to 13 years, the “cereal and milk pattern” consisting of ready-to-eat breakfast cereal, milk, and yogurt was represented by 6% of the children.

In the present study, the older age of the participants was associated with greater adherence to the “cold meats/sausages, cheeses and butter/cream” pattern, which is in line with a study carried out in Salamanca that identified an increase in the consumption of dairy products and fruits in adolescents [63]. The younger age of the participants is associated with greater adherence to the pattern “fruits/fruit juices, breakfast cereals, yogurts, and homemade cakes/pies and sweet snacks” in this study; a similar result was found in a study carried out in Malaysia, which identified a higher consumption of ready-to-eat breakfast cereals in children aged 6 to 9 years [64]. Higher maternal education was associated with greater adherence to the patterns “milk, UP bread and chocolate milk” and “fruits/fruit juices, breakfast cereals, yogurts, and homemade cakes/pies and sweet snacks” in the present study, which is in line with what was observed in the study with Brazilian children aged 8 and 9 years that found a positive association between maternal education and the pattern called “egg–dairy”, consisting of sweetened milk drinks [65]. A study carried out in the Netherlands with children aged 8 to 12 years found a positive association between fruit consumption and increased maternal education [66].

The four breakfast patterns identified in this study can be considered mixed and were composed of UP and P products, which are formulations made mainly of substances derived from food and chemical additives, containing little or no whole food; they are more energy-dense, rich in saturated fats, trans fats and added sugar, and they are low in protein, dietary fiber, and micronutrients [33]. Furthermore, UP foods are highly palatable, promoting the physiological interruption of the signs of hunger and satiety, inducing their excessive consumption, and they are associated with increased lipogenesis [67] and the accumulation of fatty acids in tissues and blood [68]. This evidence was observed in a study carried out with the same CHD children and adolescents in the present study, which found a positive association between total daily consumption of added sugar and trans fatty acids and total and central body adiposity [23]. These previous findings, associated with the results found in this study, reinforce the importance of raising awareness about the promotion of a healthy lifestyle, for the prevention of obesity and cardiovascular diseases by children and adolescents with CHD, after undergoing corrective cardiac surgery, with the introduction of healthy foods for breakfast containing whole grains, fruits, and low-fat dairy products and avoiding the consumption of ultra-processed foods.

Similar to breakfast skipping, cardiometabolic and nutritional markers were not associated with any of the four breakfast patterns in the present study. Only one study carried out in the United States [16] investigated the association between these outcomes with breakfast patterns in healthy children and adolescents through cluster analysis, which observed that children and adolescents who make up the patterns “ready-to-eat pre-sweetened cereal, low-fat milk” and “pre-sweetened cereal, whole milk” were 43% and 46%, respectively, less likely to be overweight or obese than children and adolescents who skipped breakfast [16]. In addition, a population-based study carried out with adolescents in Brazil investigated the association between breakfast patterns with weight status through principal component factor analysis, which observed that the “cereal, protein, fruit beverages and northern/northeastern” pattern was inversely associated with weight status [21]. Furthermore, foods representative of the breakfast pattern 3 “cold meats/sausages, cheeses and butter/cream” are predominantly of animal source, have a high energy density, are rich in saturated and trans fatty acids and added sugars, and are low in fiber, dietary factors considered to be risk factors for the development of obesity [23,69,70]. Although the breakfast patterns were not associated with cardiometabolic and nutritional markers in our study, breakfast skipping or breakfast patterns characterized by unhealthy foods may impact the long-term cardiovascular health of this population since they already have cardiovascular risk factors in childhood [1].

The main strengths of this study include, first, the originality of the study; second, the collection of food consumption data was obtained by three 24 h recalls on non-consecutive days; third, the sample size was representative of the population studied; finally, the use of an a posteriori approach to identify the breakfast patterns, which is a new and representative approach of the food consumption profile [71], making it possible to know the foods that are positively or negatively associated with a DP [17].

However, the present study had limitations: first, the cross-sectional design, which made it impossible to establish a causal relationship between breakfast consumption or identified breakfast patterns and cardiometabolic and nutritional markers; second, there is limited consensus as to what defines the breakfast meal. How eating occasions are defined has been shown to affect how eating patterns are quantitatively characterized. Leech et al. (2021) [30] suggested that a definition that categorizes meals and snacks using the participant identification or time-of-day approach may be a suitable choice for use in children’s DPs and obesity research; third, the lack of information that can influence breakfast composition, such as sleeping time and school hours, and housing (rural or urban); fourth, the principal component analysis presents a subjectivity of choice in the criteria for retaining the number of DPs; Lastly, the multivariable analysis between breakfast patterns and cardiometabolic and nutritional markers should be interpreted with caution, considering that the possibility of random measurement error [72,73] and stepwise selection procedure can produce biased estimates and incorrect confidence intervals [74]. However, the decisions to derive breakfast patterns as well as the selection of the variables included as potential confounders in the multivariable analysis were made according to the methodologies described in the literature [36,37,45].

## 5. Conclusions

In the present cross-sectional study in children and adolescents with CHD, a high frequency of breakfast skippers and four breakfast patterns considered mixed and composed of ultra-processed foods were identified. These findings reinforce the need to promote intervention strategies to improve the lifestyle through food and nutrition education for a healthy breakfast, promoting the reduction in the consumption of ultra-processed foods and prioritizing fresh and minimally processed foods by this population. Thus, a longitudinal study with the addition of other biomarkers or a combination of inflammatory markers could contribute to the investigation of the association between breakfast patterns and cardiometabolic and nutritional markers.

## Figures and Tables

**Table 1:** Food groups included in the analysis of breakfast patterns in children and adolescents with CHD. Brazil (2017).

Degree of Processing	Food Groups	Description of Food
Fresh or minimally processed foods and culinary ingredients	Milks	Milk; milk-based fruit vitamins (whole, semi and skimmed)
	Butter and cream	Butter and cream
	Fruits	Orange; banana; apple; avocado; pineapple among other fresh fruits and fruit salad. Natural fruit juice (various flavors) and natural coconut water
	Homemade cakes/pies and pastries	Homemade cakes/pies (carrot cake, cassava cake, pie in various flavor and others), homemade Brazilian sweets (tapioca pearls in red wine, waffles, creamy hominy pudding), arrowroot biscuit, butter cookie and oatmeal cookie
	Coffee and tea	Coffee and tea
	Sugars	White; demerara and muscovado
Processed foods	Breads	Wheat bread; cornbread; rye bread; chestnut bread; Italian bread; baguette; homemade toast
	Cheese	Parmesan and cheeses in general with high fat and low-fat content
Ultra-processed	Breads	Industrialized loaf; hot dog bread, hamburger bread; biscuits; sprinkles; garlic bread; flatbread
	Cold meats	Bacon, salami, hot dog sausage, ham, pate, crackling
	Margarines	Margarine (salted and unsalted)
	Salted crackers	Salted crackers with and without filling
	Breakfast cereals	Breakfast cereals; industrialized granola and dairy flour
	Sweetening products	Traditional Brazilian sweets (brigadeiro, beijinho, casadinho, maria mole), processed cakes and pastries (cakes, sweet muffins) desserts (puddings, mousse, gelatin), condensed milk, cakes made mostly with ultra-processed products, processed flavors yogurt, sugar spreads (jam, dulce de leche and hazelnut cream) ice cream and popsicle (various flavors),
	Ready to eat and take-away/fast foods	Calzones; baked pastries; esfiha; puffs pastries; cheese breads; instant soups; sandwich hot dog or meat sandwich; fritters snacks; Brazilian snack (pastel; coxinha; risolis); pizza (various flavors, with and without border; French fries; sauces (mayonnaise; ketchup; mustard); canned vegetables (cucumber; corn; olives; peas); chips; microwave popcorn
	Biscuits	Biscuits (with filling and without filling)
	Yogurts	Yogurts in general (with high fat and low fat)
	Chocolate	Chocolate powder
	Other sugary drinks and soft drinks	Sweetened beverages artificial juice and tea; soft drinks

**Table 2:** Sociodemographic, clinical and lifestyle data of a cross-sectional study with 232 children and adolescents with CHD according to breakfast consumption. Brazil (2017).

Variables	Total	Breakfast Consumption		
	n = 232	No n = 63 (27%)	Yes n = 169 (73%)	*p* Value
	n (%) or (Mean ± SD) ^a^	n (%) or (Mean ± SD) ^a^		
Age (years)	10.2 ± 3.7	10.77 ± 3.5	10 ± 3.9	0.16 ^b^
Sex				0.40 ^c^
Female	122 (52.5)	36 (57.1)	86 (50.9)	
Male	110 (47.5)	27 (42.9)	83 (49.1)	
Mother’s education				0.61 ^c^
<10 years of schooling	99 (43)	25 (40.4)	74 (44)	
≥10 years of schooling	131 (57)	37 (59.6)	94 (56)	
Sedentary behavior				0.11 ^c^
No (<8 h/day)	85 (36.6)	18 (28.5)	67 (39.6)	
Yes (≥8 h/day)	147 (63.4)	45 (71.5)	102 (60.4)	
Postoperative time (years)	6.7 ± 3.8	7.12 ± 3.71	6.60 ± 3.88	0.33 ^b^
Family history for obesity				0.001 ^c^
Absent	147 (64.5)	30 (47.6)	117 (71)	
Present	81 (35.5)	33 (52.4)	48 (29)	
Congenital heart disease				0.01 ^c^
Cyanotic	79 (34)	21 (33.4)	58 (34.4)	
Acyanotic	153 (66)	42 (66.6)	111 (65.6)	

Legend: %, percentages; n, absolute number; ^a^ SD, standard deviation; ^b^ Student’s *t* test; ^c^ Chi-square test.

**Table 3:** Breakfast patterns and rotational factor loadings of the identified food groups of children and adolescents with CHD (n = 169). Brazil (2017).

Food Groups	Milk, Bread UP ^a^ and Chocolate Milk	Margarine and Bread P ^b^	Cold Meats/Sausages, Cheeses and Butter/Cream	Fruits/Fruit Juices, Breakfast Cereals, Yogurts and Homemade Cakes/Pies and Sweets Snacks
Milks	0.52	-	-	-
Breads UP ^a^	0.48	-	-	-
Chocolate	0.47	-	-	-
Homemade cakes/pies and pastries	−0.28	-	-	0.26
Coffee and tea	−0.35	-	-	−0.30
Margarine	-	0.59	-	-
Breads P ^b^	-	0.51	-	-
Cold cuts and processed meats	-	-	0.48	-
Cheeses	-	-	0.47	-
Butter and cream	-	-	0.44	-
Sugary drinks and soft drinks	-	-	−0.28	-
Fruits	-	-	-	0.39
Breakfast cereals	-	-	-	0.37
Yoghurt	-	-	-	0.27
Sugar	-	-	-	−0.59
Salted crackers	-	-	-	-
Sweetening products	-	-	-	-
Ready to eat and take-away/fast foods	-	-	-	-
Biscuits	-	-	-	-
Variability (%)	11.2	9.3	8.6	8.0
Accumulated variability (%)	37.0			
* KMO value	0.55			

* KMO value = value = Kaiser–Meyer–Olkin statistical test. Factorial loads |≥0.25| significant contribution to breakfast patterns. Legend: ^a^ UP, ultra-processed; ^b^ P, processed.

**Table 4:** Sociodemographic, clinical and lifestyle data of a cross-sectional study with 169 children and adolescents with CHD according to the classification of below and above the median of each breakfast pattern. Brazil (2017).

	Breakfast Patterns							
Variables	Milk, Bread UP ^a^ and Chocolate Milk		Margarine and Bread P ^b^		Cold Meats, Cheeses and Butter/Cream		Fruits/Fruit Juices, Breakfast Cereals, Yogurts and Homemade Cakes/Pies and Sweet Snacks	
	Below the Median	Above the Median	Below the Median	Above the Median	Below the Median	Above the Median	Below the Median	Above the Median
	n (%) or (Mean ± SD) ^c^							
Age (years)	11.2 ± 4.1	8.8 ± 3.2	9.7 ± 3.1	10.2 ± 3.7	9.1 ± 4	10.8 ± 3.5	11.1 ± 4	8.8 ± 3.5
Value *p* ^d^	0.001		0.35		0.003		0.001	
Sex								
Female	45 (53)	41 (49)	46 (54.1)	40 (47.6)	41 (48.2)	45 (53.6)	42 (49.4)	44 (52.4)
Male	40 (47)	43 (51)	39 (45.9)	44 (52.4)	44 (51.8)	39 (46.4)	43 (50.6)	40 (47.6)
Value *p* ^e^	0.60		0.60		0.48		0.70	
Mother’s education (years of schooling)								
<10	25 (29.8)	12 (14.3)	13 (15.3)	24 (28.9)	18 (21.2)	19 (22.9)	27 (31.7)	10 (12)
≥10	59 (70.2)	72 (85.7)	72 (84.7)	59 (71.1)	67 (78.8)	64 (77.1)	58 (68.2)	73 (88)
Value *p* ^e^	0.01		0.13		0.78		0.002	
Sedentary behavior								
No	34 (40)	33 (39.3)	31 (36.5)	36 (42.9)	36 (42.4)	31 (37)	31 (36.5)	36 (42.9)
Yes	51 (60)	51 (60.7)	54 (63.5)	48 (57.1)	49 (57.6)	53 (63)	54 (63.5)	48 (57.1)
Value *p* ^e^	0.92		0.39		0.46		0.39	
Postoperative time (years)	7 ± 4.4	6 ± 3.2	6.1 ± 3.8	7 ± 3.8	6 ± 46	7.1 ± 3.7	7 ± 4.2	6.1 ± 3.4
Value *p* ^d^	0.08		0.14		0.04		0.12	
Family history for obesity								
Absent	59 (70.2)	58 (71.6)	62 (74.7)	55 (67)	60 (70.6)	57 (71.2)	54 (66)	63 (76)
Present	25 (29.8)	23 (28.4)	21 (25.3)	27 (33)	25 (29.4)	23 (28.8)	28 (34)	20 (24)
Value *p* ^e^	0.84		0.28		0.92		0.15	
Congenital heart disease								
Cyanotic	27 (31.8)	31 (37)	30 (35.3)	28 (33.3)	30 (35.3)	28 (33.3)	29 (34)	29 (34.5)
Acyanotic	58 (68.2)	53 (63)	55 (64.7)	56 (66.7)	55 (64.7)	56 (66.7)	56 (66)	55(65.5)
Value *p* ^e^	0.48		0.78		0.78		0.95	

Legend: ^a^ UP, ultra-processed; ^b^ P, processed; ^c^ SD, standard deviation; ^d^ Student’s *t* test; ^e^ Chi-square test.

**Table 5:** Cross-sectional associations between skipping breakfast and cardiometabolic and nutritional parameters of children and adolescents with CHD. Brazil (2017).

	Skipping Breakfast	
	β ^a^ (CI 95% ^b^)	*p*
Total cholesterol (mg/dL) (n = 229)		
Unadjusted	−0.72 (−8.38–6.92)	0.85
Adjusted 1	1.74 (−10.92–7.43)	0.70
Adjusted 2	3.20 (−7.45–13.87)	0.55
HDL-c (mg/dL) ^c^ (n = 232)		
Unadjusted	1.94 (−1.72–5.61)	0.29
Adjusted 1	2.74 (−1.60–7.09)	0.21
Adjusted 2	3.32 (−1.74–8.38)	0.19
Non-HDL-c (mg/dL) (n = 227)		
Unadjusted	−3.32 (−10.25–3.61)	0.34
Adjusted 1	−5.23 (−13.44–2.97)	0.21
Adjusted 2	−5.99 (−10.29–8.30)	0.18
LDL-c (mg/dL) ^d^ (n = 230)		
Unadjusted	−0.96 (−7.70–5.77)	0.77
Adjusted 1	−2.74 (−10.57–5.09)	0.49
Adjusted 2	−3.08 (−8.92–6.76)	0.39
Triglycerides (mg/dL) (n = 225)		
Unadjusted	−1.80 (−8.98–5.38)	0.39
Adjusted 1	−3.37 (−11.86–5.11)	0.43
Adjusted 2	−1.31 (−10.95–8.31)	0.78
Glucose (mg/dL) (n = 227)		
Unadjusted	−0.73 (−2.52–1.06)	0.42
Adjusted 1	−0.86 (−2.90–1.16)	0.40
Adjusted 2	−0.13 (−2.54–2.28)	0.39
hs-CRP (mg/dL) ^e^ (n = 220)		
Unadjusted	0.02 (−0.15–0.20)	0.77
Adjusted 1	−0.02 (−0.22–0.17)	0.82
Adjusted 2	−0.01 (−0.23–0.21)	0.93
Systolic blood pressure (percentile) (n = 232)		
Unadjusted	−0.80 (−7.48–5.87)	0.81
Adjusted 1	−1.09 (−11.14–4.95)	0.44
Adjusted 2	−1.67 (−17.73–5.39)	0.36
Diastolic blood pressure (percentile) (n = 229)		
Unadjusted	−0.42 (−5.13–4.29)	0.86
Adjusted 1	−1.85 (−7.47–3.77)	0.51
Adjusted 2	−1.95 (−8.23–2.42)	0.22
Waist circumference (percentile) (n = 229)		
Unadjusted	2.67 (−0.29–5.63)	0.10
Adjusted 1	2.60 (−0.70–5.92)	0.12
Adjusted 2	2.32 (−1.41–6.05)	0.22
Lean mass (%) (n = 230)		
Unadjusted	−2.32 (−4.92–0.27)	0.10
Adjusted 1	−2.79 (−5.80–0.20)	0.08
Adjusted 2	−3.13 (−6.63–0.36)	0.07
Body fat (%) (n = 230)		
Unadjusted	2.26 (−0.32–4.85)	0.10
Adjusted 1	2.71 (−0.26–5.68)	0.08
Adjusted 2	2.88 (−0.61–5.85)	0.07

Reference: Participants who eat breakfast; Variables transformed into log = triglycerides (mg/dL), glucose (mg/dL), hs-CRP (mg/dL), systolic blood pressure (percentile) and WC (percentile), values presented in log. Legend: ^a^ β, beta-coefficients; ^b^ CI 95%, 95% confidence interval; ^c^ HDL-c, high density lipoprotein cholesterol; ^d^ LDL-c, low-density lipoprotein cholesterol; ^e^ hs-CRP, high sensitivity c-reactive protein; Adjusted 1, adjusted for age (in years), gender (female/male), mother’s education (years); Adjusted 2, adjusted 1 + sedentary behavior (in hours), postoperative time (years), family history for obesity (absent/present), classification of congenital heart disease (according to the international code of diseases (ICD-10)), waist circumference (percentile) and total daily energy (kcal/ day); Total cholesterol (n = 3 missing data); HDL-c (n = 1 missing date); non-HDL-c (n = 5 missing data); LDL-c (n = 2 missing data); triglycerides (n = 7 missing data); glucose (n = 5 missing data); hs-CRP (n = 12 missing data); SBP (n = 2 missing data); DBP (n = 3 missing data); WC (n = 3 missing data); lean mass (n = 2 missing data) and body fat (n = 2 missing data).

**Table 6:** Cross-sectional associations between cardiometabolic parameters and breakfast patterns of 169 children and adolescents with CHD. Brazil (2017).

	Breakfast Patterns (Z Score)							
	Milk, Bread UP ^a^, Chocolate Milk		Margarine and Bread P ^b^		Cold Meats/Sausages, Cheeses and Butter/Cream		Fruits/Fruit Juices, Breakfast Cereals, Yogurts and Homemade Cakes/Pies and Sweet Snacks	
	β ^c^ (CI 95%) ^d^	*p*	β (CI 95%)	*p*	β (CI 95%)	*p*	β (CI 95%)	*p*
Total cholesterol (mg/dL) (n = 167)								
Unadjusted	0.57 (−2.16–3.32)	0.67	0.65 (−2.39–3.70)	0.67	0.28 (−2.85–3.43)	0.85	1.62 (−1.65–4.91)	0.32
Adjusted 1	−0.31 (−3.24–2.60)	0.83	1.27 (−1.80–4.35)	0.41	0.74 (−2.38–3.87)	0.63	1.02 (−2.33–4.39)	0.54
Adjusted 2	−0.38 (−3.38–2.61)	0.80	−0.38 (−3.38–2.61)	0.80	0.68 (−2.51–3.88)	0.67	0.93 (−2.57–4.44)	0.60
HDL-c (mg/dL) ^e^ (n = 168)								
Unadjusted	0.80 (−0.46–2.08)	0.21	−1.04 (−2.45–0.36)	0.14	−0.35 (−1.81–1.10)	0.63	−0.05 (−1.60–1.48)	0.94
Adjusted 1	0.35 (−1.02–1.73)	0.61	−0.78 (−2.22–0.65)	0.28	−0.10 (−1.57–1.36)	0.88	−0.39 (−1.97–1.20)	0.62
Adjusted 2	0.50 (−0.87–1.88)	0.47	−0.21 (−1.76–1.34)	0.78	0.27 (−1.19–1.74)	0.71	−0.33 (−1.95–1.28)	0.68
Adjusted 3	0.58 (−0.90–1.87)	0.48	−0.18 (−1.74–1.37)	0.81	0.28 (−1.19–1.75)	0.70	−0.33 (−1.95–1.30)	0.68
Non-HDL-c (mg/dL) (n = 166)								
Unadjusted	−0.01 (−2.44–2.41)	0.99	2.03 (−0.64–4.72)	0.13	0.30 (−2.50–3.10)	0.82	1.90 (−1.00–4.80)	0.19
Adjusted 1	−0.16 (−2.80–2.46)	0.90	2.35 (−0.39–5.10)	0.09	0.40 (−2.43–3.22)	0.78	1.76 (−1.25–4.77)	0.25
Adjusted 2	−0.40 (−3.08–2.27)	0.76	1.45 (−1.57–4.47)	0.34	−0.01 (−2.88–2.85)	0.99	1.60 (−1.51–4.72)	0.31
Adjusted 3	−0.27 (−2.94–2.40)	0.84	1.30 (−1.71–4.32)	0.40	−0.07 (−2.93–2.78)	0.95	1.60 (−1.51–4.71)	0.31
LDL-c (mg/dL) ^f^ (n = 168)								
Unadjusted	0.06 (−2.30–2.41)	0.95	2.04 (−0.54–4.62)	0.12	−0.11 (−2.80–2.60)	0.93	1.27 (−1.52–4.06)	0.36
Adjusted 1	0.10 (−2.43–2.65)	0.93	2.32 (−0.32–4.96)	0.08	−0.08 (−2.81–2.64)	0.95	1.32 (−1.56–4.21)	0.36
Adjusted 2	0.25 (−2.83–2.32)	0.84	1.35 (−1.53–4.24)	0.35	−0.43 (−3.20–2.31)	0.75	1.16 (−1.81–4.14)	0.44
Triglycerides (mg/dL) (n = 167)								
Unadjusted	0.97 (0.93–1.01)	0.24	1.02 (0.96–1.07)	0.44	1.00 (0.94–1.05)	0.91	0.97 (0.92–1.03)	0.45
Adjusted 1	0.97 (0.92–1.02)	0.27	1.02 (0.96–1.07)	0.44	1.00 (0.94–1.05)	0.88	0.97 (0.92–1.03)	0.39
Adjusted 2	0.97 (0.92–1.02)	0.31	1.01 (0.95–1.07)	0.66	1.00 (0.94–1.05)	0.84	0.97 (0.92–1.03)	0.40
Glucose (mg/dL) (n = 165)								
Unadjusted	1.00 (0.98–1.00)	0.38	1.00 (1.00–1.01)	0.10	1.00 (1.00–1.01)	0.12	1.00 (0.98–1.00)	0.70
Adjusted 1	1.00 (0.98–1.00)	0.37	1.00 (0.99–1.01)	0.35	1.00 (0.99–1.01)	0.34	1.00 (0.99–1.00)	0.80
Adjusted 2	1.00 (0.98–1.00)	0.30	1.00 (0.99–1.01)	0.31	1.00 (0.99–1.01)	0.33	1.00 (0.99–1.00)	0.92
hs-CRP (mg/L) ^g^ (n = 157)								
Unadjusted	1.03 (0.96–1.10)	0.32	1.02 (0.94–1.10)	0.57	1.01 (0.94–1.10)	0.62	1.12 (1.03–1.21)	0.42
Adjusted 1	1.03 (0.96–1.10)	0.36	1.01 (0.94–1.09)	0.66	1.01 (0.94–1.10)	0.63	1.12 (1.03–1.22)	0.42
Adjusted 2	1.02 (0.94–1.09)	0.52	0.98 (0.90–1.06)	0.66	1.02 (0.93–1.09)	0.73	1.14 (1.05–1.24)	0.40
SBP (percentile) ^h^ (n = 167)								
Unadjusted	1.01 (0.93–1.10)	0.78	0.94 (0.86–1.02)	0.18	1.00 (0.91–1.10)	0.97	1.07 (0.98–1.18)	0.11
Adjusted 1	0.98 (0.90–1.06)	0.66	0.94 (0.88–1.05)	0.20	1.00 (0.91–1.10)	0.91	1.06 (0.96–1.17)	0.21
Adjusted 2	0.98 (0.90–1.07)	0.66	0.94 (0.85–1.04)	0.24	1.00 (0.91–1.10)	0.91	1.06 (0.96–1.17)	0.21
DBP (percentile) ^i^ (n = 167)								
Unadjusted	1.44 (−0.22–3.11)	0.09	1.35 (−1.48–2.20)	0.70	0.56 (−1.39–2.33)	0.57	1.10 (−0.87–3.10)	0.27
Adjusted 1	1.40 (−1.69–1.76)	0.11	1.17 (−0.62–2.97)	0.20	0.46 (−1.39–2.33)	0.61	0.30 (−1.65–2.25)	0.76
Adjusted 2	1.35 (−1.69–1.76)	0.11	1.17 (−0.62–2.97)	0.20	0.46 (−1.39–2.33)	0.63	0.30 (−1.65–2.25)	0.76
WC (percentile) ^j,k^ (n = 160)								
Unadjusted	1.00 (0.94–1.05)	0.91	1.02 (0.95–1.08)	0.48	1.06 (1.00–1.13)	0.16	1.02 (0.95–1.10)	0.51
Adjusted 1	0.97 (0.92–1.04)	0.48	1.04 (0.97–1.11)	0.21	1.06 (1.00–1.13)	0.16	1.00 (0.93–1.07)	0.53
Adjusted 2 ^k^	0.98 (0.92–1.04)	0.69	1.04 (0.98–1.12)	0.16	1.07 (1.00–1.14)	0.18	1.00 (0.93–1.07)	0.54
Body fat (%) (n = 168)								
Unadjusted	−0.14 (−1.27–0.44)	0.34	−0.26 (−1.23–0.70)	0.60	0.40 (−0.57–1.35)	0.42	0.18 (−0.81–1.17)	0.71
Adjusted 1	−0.14 (−1.27–0.43)	0.34	−0.26 (−1.23–0.70)	0.57	0.22 (−0.72–1.16)	0.64	0.14 (−0.85–1.17)	0.77
Adjusted 2	−0.13 (−0.91–0.64)	0.35	−0.33 (−1.17–0.50)	0.47	0.21 (−0.72–1.17)	0.65	0.13 (−0.85–1.16)	0.78
Lean mass (%) (n = 168)								
Unadjusted	0.38 (−0.47–1.24)	0.37	0.21 (−0.76–1.20)	0.66	−0.41 (−1.40–0.55)	0.40	−0.16 (−1.16–0.83)	0.74
Adjusted 1	0.36 (−0.47–1.25)	0.37	0.22 (−0.76–1.19)	0.65	−0.42 (−1.40–0.56)	0.38	−0.14 (−1.15–0.86)	0.78
Adjusted 2	0.35 (−0.46–1.28)	0.38	0.23 (−0.76–1.19)	0.64	−0.42 (−1.40–0.56)	0.38	−0.10 (−1.11–0.90)	0.80

Legend: ^a^ UP, ultra-processed; ^b^ P, processed; ^c^ β, beta-coefficients; ^d^ CI 95%, 95% confidence interval; ^e^ HDL-c, high density lipoprotein cholesterol; ^f^ LDL-c, low-density lipoprotein cholesterol; ^g^ hs-CRP, high sensitivity c-reactive protein; ^h^ SBP, systolic blood pressure; ^i^ DBP, diastolic blood pressure; ^j^ WC, waist circumference. Adjusted 1, adjusted for age (in years), sex (female/male), mother’s education (<10 years and ≥10 years); Adjusted 2, adjusted by adjusted 1 + sedentary behavior (in hours), postoperative time (in years), family history for obesity (no/yes), congenital heart disease (cyanotic and acyanotic) and WC (percentile); ^k^ Variable was not adjusted for WC (percentile); Adjusted 3, adjusted by adjusted 2 + Glucose (mg/dL). Variables transformed into log = triglycerides (mg/dL), glucose (mg/dL), hs-CRP (mg/dL), SBP (percentile) and WC (percentile), values presented in log. Total cholesterol (n = 2 missing data); HDL-c (n = 1 missing date); non-HDL-c (n = 3 missing data); LDL-c (n = 1 missing date); triglycerides (n = 2 missing data); glucose (n = 4 missing data); hs-CRP (n = 12 missing data); SBP (n = 2 missing data); DBP (n = 2 missing data); WC (n = 9 missing data); body fat (n = 1 missing data) and lean mass (n = 1 missing data).

## Data Availability

The data presented in this study are available on request from the corresponding author. The data are not publicly available due to privacy.

## Supplementary Materials

The following supporting information can be downloaded at: https://www.mdpi.com/article/10.3390/ijerph20065146/s1, Figure S1: Flowchart of the selection of children and adolescents with CHD. Florianópolis, Southern Brazil, 2017.

## References

[1] Tamayo C., Manlhiot C., Patterson K., Lalani S., McCrindle B.W. (2015). Longitudinal evaluation of the prevalence of overweight/obesity in children with congenital heart disease. Can. J. Cardiol..

[2] Cheuk D.K., Wong S.M.Y., Choi Y.P., Chau A.K.T., Cheung Y.F. (2004). Parents’ understanding of their child’s congenital heart disease. Heart.

[3] Uzark K., Jones K., Slusher J., Limbers C.A., Burwinkle T.M., Varni J.W. (2008). Quality of life in children with heart disease as perceived by children and parents. Pediatrics.

[4] Massin M.M., Hövels-Gürich H., Seghaye M.C. (2007). Atherosclerosis lifestyle risk factors in children with congenital heart disease. Eur. J. Prev. Cardiol..

[5] Cohen M.S. (2012). Clinical practice: The effect of obesity in children with congenital heart disease. Eur. J. Pediatr..

[6] Hoffman J.L., Mack G.K., Minich L.L., Benedict S.L., Heywood M., Stoddard G.J., Saarel E.V. (2011). Failure to impact prevalence of arterial ischemic stroke in pediatric cardiac patients over three decades. Congenit. Heart Dis..

[7] Funtikova A.N., Navarro E., Bawaked R.A., Fíto M., Schröder H. (2015). Impact of diet on cardiometabolic health in children and adolescents. Nutr. J..

[8] Arenaza L., Muñoz-Hernández V., Medrano M., Oses M., Amasene M., Merchán-Ramírez E., Cadenas-Sanchez C., Ortega F.B., Ruiz J.R., Labayen I. (2018). Association of breakfast quality and energy density with cardiometabolic risk factors in overweight/obese children: Role of physical activity. Nutrients.

[9] O’Neil C.E., Byrd-Bredbenner C., Hayes D., Jana L., Klinger S.E., Stephenson-Martin S. (2014). The role of breakfast in health: Definition and criteria for a quality breakfast. J. Acad. Nutr. Diet..

[10] Chang Y., Gable S. (2013). Predicting weight status stability and change from fifth grade to eighth grade: The significant role of adolescents social-emotional well-being. J. Adolesc. Health.

[11] Quick V., Wall M., Larson N., Haines J., Neumark-Sztainer D. (2013). Personal, behavioral and socio-environmental predictors of overweight incidence in young adults: 10-yr longitudinal findings. Int. J. Behav. Nutr. Phys. Act..

[12] Monzani A., Rapa A., Fuiano N., Diddi G., Prodam F., Bellone S., Bona G. (2014). Metabolic syndrome is strictly associated with parental obesity beginning from childhood. Clin. Endocrinol..

[13] Ho C.Y., Huang Y.C., Lo Y.T.C., Wahlqvist M.L., Lee M.S. (2015). Breakfast is associated with the metabolic syndrome and school performance among Taiwanese children. Res. Dev. Disabil..

[14] Rosato V., Edefonti V., Parpinel M., Milani G.P., Mazzocchi A., Decardi A., Agostini C., Ferraroni M. (2016). Energy contribution and nutrient composition of breakfast and their relations to overweight in free-living individuals: A systematic review. Adv. Nutr..

[15] Afeiche M.C., Taillie L.S., Hopkins S., Eldridge A.L., Popkin B.M. (2017). Breakfast dietary patterns among Mexican children are related to total-day diet quality. J. Nutr..

[16] O’Neil C.E., Nicklas T.A., Fulgoni V.L. (2015). Nutrient intake, diet quality, and weight measures in breakfast patterns consumed by children compared with breakfast skippers: NHANES 2001–2008. AIMS Public Health.

[17] Ambrosini G.L. (2014). Childhood dietary patterns and later obesity: A review of the evidence. Proc. Nutr. Soc..

[18] O’Neil C.E., Nicklas T.A., Fulgoni V.L. (2014). Nutrient intake, diet quality, and weight/adiposity parameters in breakfast patterns compared with no breakfast in adults: National Health and Nutrition Examination Survey 2001–2008. J. Acad. Nutr. Diet..

[19] Iqbal K., Schwingshackl L., Gottschald M., Knüppel S., Stelmach-Mardas M., Aleksandrova K., Boeing H. (2017). Breakfast quality and cardiometabolic risk profiles in an upper middle-aged German population. Eur. J. Clin. Nutr..

[20] Chatelan A., Castetbon K., Pasquier J., Allemann C., Zuber A., Camenzind-Frey E., Zuberbuehler C.A., Bochud M. (2018). Association between breakfast composition and abdominal obesity in the Swiss adult population eating breakfast regularly. Int. J. Behav. Nutr. Phys. Act..

[21] Hassan B.K., Cunha D.B., Santos R.O., Baltar V.T. (2021). Breakfast patterns and weight status among adolescents: A study on the Brazilian National Dietary Survey 2008–2009. Br. J. Nutr..

[22] Honicky M., Souza J.N., Cardoso S.M., de Carlos Back I., Vieira F.G.K., de Fragas Hinnig P., Moreno Y.M.F. (2021). Dietary patterns are associated with central adiposity and carotid intima-media thickness in children and adolescents with congenital heart disease. Eur. J. Nutr..

[23] Honicky M., Cardoso S.M., Lima L.R.A., Ozariz S.G.I., Vieira F.G.K., de Carlos Back I., Moreno I.M.F. (2020). Added sugar and trans fatty acid intake and sedentary behavior were associated with excess total-body and central adiposity in children and adolescents with congenital heart disease. Pediatr. Obes..

[24] Conway J.M., Ingwersen L.A., Moshfegh A.J. (2004). Accuracy of dietary recall using the USDA five-step multiple-pass method in men: An observational validation study. J. Am. Diet. Assoc..

[25] Zabotto C.B., Vianna R.P., Gil M.F. (1996). Registro Fotográfico para Inquéritos Dietéticos: Utensílios e Porções.

[26] Núcleo de Estudos e Pesquisas em Alimentação, Universidade Estadual de Campinas (2011). Tabela Brasileira de Composição de Alimentos.

[27] Fisberg R.M., Marchioni D.M.L. (2002). Manual de Receitas e Medidas Caseiras para Cálculo de Inquéritos Alimentares: Manual Elaborado para Auxiliar no Procedimento de Inquéritos Alimentares.

[28] Bombem K.C.M. (2012). Manual de Medidas Caseiras e Receitas para Cálculos Dietéticos.

[29] Pinheiro A.B. (1998). Tabela para Avaliação de Consumo Alimentar em Medidas Caseiras.

[30] Leech R.M., Spence A.C., Lacy K.E., Zheng M., Timperio A., McNaughton S.A. (2021). Characterizing children’s eating patterns: Does the choice of eating occasion definition matter?. Int. J. Behav. Nutr. Phys. Act..

[31] Cheng T.S.Y., Tse L.A., Yu I.T.S., Griffiths S. (2008). Children’s perceptions of parental attitude affecting breakfast skipping in primary sixth-grade students. J. Sch. Health.

[32] Dubois L., Girard M., Kent M.P., Farmer A., Tatone-Tokuda F. (2009). Breakfast skipping is associated with differences in meal patterns, macronutrient intakes and overweight among pre-school children. Public Health Nutr..

[33] Monteiro C.A., Cannon G., Moubarac J.C., Levy R.B., Louzada M.L.C., Jaime P.C. (2018). The UN Decade of Nutrition, the NOVA food classification and the trouble with ultra-processing. Public Health Nutr..

[34] Willett W., Stampfer M.J. (1986). Total energy intake: Implications for epidemiologic analyses. Am. J. Epidemiol..

[35] Hair J.F., Black W.C., Babin B.J., Anderson R.E. (2009). Multivariate Data Analysis.

[36] Olinto M.T.A., Kac G., Sichieri R., Gigante D.P. (2007). Dietary patterns: Analysis of components. Nutritional Epidemiology.

[37] Liu S., Joseph K.S., Luo W., León J.A., Lisonkova S., Van den Hof M., Evans J., Lim K., Little J., Sauve R. (2016). Effect of Folic Acid Food Fortification in Canada on Congenital Heart Disease Subtypes. Circulation.

[38] Van Der Ploeg H.P., Chey T., Korda R.J., Banks E., Bauman A. (2012). Sitting time and all-cause mortality risk in 222,497 Australian adults. Arch. Intern. Med..

[39] National Health and Nutrition Examination Survey (NHANES)—National Center for Health Statistics (NCHS) Anthropometry Procedures Manual. Hyattsville, Maryland, USA, 2002. https://www.cdc.gov/nchs/data/nhanes/bm.pdf.

[40] Sharma A.K., Metzger D.L., Daymont C., Hadjiyannakis S., Rodd C.J. (2015). LMS tables for waist-circumference and waist-height ratio Z-scores in children aged 5–19 y in NHANES III: Association with cardio-metabolic risks. Pediatr. Res..

[41] Dempster P., Aitkens S. (1995). A new air displacement method for the determination of human body composition. Med. Sci. Sport. Exerc..

[42] Lohman T.G. (1989). Assessment of body composition in children. Pediatr. Exerc. Sci..

[43] Friedewald W.T., Levy R.I., Fredrickson D.S. (1972). Estimation of the concentration of low-density lipoprotein cholesterol in plasma, without use of the preparative ultracentrifuge. Clin. Chem..

[44] U.S. Department of Health and Human Services, National Institutes of Health, National Heart, Lung and Blood Institute The Fourth Report on the Diagnosis, Evaluation, and Treatment of High Blood Pressure in Children and Adolescents. Bethesda, Maryland, USA. https://www.nhlbi.nih.gov/sites/default/files/media/docs/hbp_ped.pdf.

[45] Chen C.A., Wang J.K., Lue H.C., Hua Y.C., Chang M.H., Wu M.H. (2012). A shift from underweight to overweight and obesity in Asian children and adolescents with congenital heart disease. Paediatr. Perinat. Epidemiol..

[46] Dodd K.W., Guenther P.M., Freedman L.S., Subar A.F., Kipnis V., Midthune D., Tooze J.A., Krebs-Smith S.M. (2006). Statistical methods for estimating usual intake of nutrients and foods: A review of the theory. J. Am. Diet. Assoc..

[47] Mota C.H., Mastroeni S.S.D.B.S., Mastroeni M.F. (2013). School meal consumption on municipal school. Rev. Bras. Estud. Pedagog..

[48] Leal G.V.S., Philippi S.T., Matsudo S.M.M., Toassa E.C. (2010). Food intake and meal patterns of adolescents, São Paulo, Brazil. Rev. Bras. Epidemiol..

[49] Pereira J.L., Castro M.A., Hopkins S., Gugger C., Fisberg R.M., Fisberg M. (2018). Prevalence of consumption and nutritional content of breakfast meal among adolescents from the Brazilian National Dietary Survey. J. Pediatr..

[50] Albashtawy M. (2017). Breakfast eating habits among schoolchildren. J. Pediatr. Nurs..

[51] Ramsay S.A., Bloch T.D., Marriage B., Shriver L.H., Spees C.K., Taylor C.A. (2018). Skipping breakfast is associated with lower diet quality in young US children. Eur. J. Clin. Nutr..

[52] Fiuza R.F.P., Muraro A.P., Rodrigues P.R.M., Sena E.M.S., Ferreira M.G. (2017). Skipping breakfast and associated factors among Brazilian adolescents. Rev. Nutr..

[53] Marchioni D.M.L., Gorgulho B.M., Teixeira J.A., Júnior Verly E., Fisberg R.M. (2015). Prevalence of breakfast omission and associated factors among adolescents in São Paulo: ISA-Capital. Nutrire Rev. Soc. Bras. Aliment. Nutr..

[54] Hassan N.E., El Shebini S.M., Ahmed N.H. (2016). Association between dietary patterns, breakfast skipping and familial obesity among a sample of Egyptian families. Open Access Maced. J. Med. Sci..

[55] Alexander K.E., Ventura E.E., Spruijt-Metz D., Weigensberg M.J., Goran M.I., Davis J.N. (2009). Association of breakfast skipping with visceral fat and insulin indices in overweight Latino youth. Obesity.

[56] Küpers L.K., Pijper J.J., Sauer P.J.J., Stolk R.P., Corpeleijn E. (2014). Skipping breakfast and overweight in 2- and 5-year-old Dutch children—The GECKO Drenthe cohort. Int. J. Obes..

[57] Champilomati G., Notara V., Prapas C., Konstantinou E., Kordoni M., Velentza A., Mesimeri M., Antonogeorgos G., Rojas-Gil A.P., Kornilaki E.N. (2020). Breakfast consumption and obesity among preadolescents: An epidemiological study. Pediatr. Int..

[58] Ruiz E., Avila J.M., Valero T., Rodriguez P., Varela-Moreiras G. (2018). Breakfast consumption in Spain: Patterns, nutrient intake and quality. Findings from the ANIBES Study, a study from the international breakfast research initiative. Nutrients.

[59] Silva F.A., Padez C., Sartorelli D.S., Oliveira R.M.S., Netto M.P., Mendes L.L., Cândido A.P.C. (2018). Cross-sectional study showed that breakfast consumption was associated with demographic, clinical and biochemical factors in children and adolescents. Acta Paediatr..

[60] Lepicard E.M., Maillot M., Vieux F., Viltard M., Bonnet F. (2017). Quantitative and qualitative analysis of breakfast nutritional composition in French schoolchildren aged 9–11 years. J. Hum. Nutr. Diet..

[61] Monteiro L.S., Souza A.M., Hassan B.K., Estima C.C.P., Sichieri R., Pereira R.A. (2017). Breakfast eating among Brazilian adolescents: Analysis of the National Dietary Survey 2008–2009. Rev. Nutri..

[62] Cezimbra V.G., Assis M.A.A., Oliveira M.T., Pereira L.J., Vieira F.G.K., Di Pietro P.F., Roberto D.M.T., Geraldo A.P.G., Soar C., Rockenbach G. (2020). Meal and snack patterns of 7–13-year-old schoolchildren in southern Brazil. Public Health Nutr..

[63] Guevara R.M., Urchaga J.D., Cabaco A.S., Moral-Garcia J.E. (2020). The quality of breakfast and healthy diet in school-aged adolescents and their association with BMI, weight loss diets and the practice of physical activity. Nutrients.

[64] Nasir M.T.M., Nurliyana A.R., Norimah A.K., Mohamed H.J.B.J., Tan S.Y., Appukutty M., Hopkins S., Thielecke F., Ong M.K., Ning C. (2017). Consumption of ready-to-eat cereals (RTEC) among Malaysian children and association with socio-demographics and nutrient intakes—Findings from the My Breakfast study. Food Nutr. Res..

[65] Vila J.K.D., Silva A.R.E., Santos T.S.S., Ribeiro A.Q., Silva A.R., Pessoa M.C., Sant’Ana L.F.R. (2015). Dietary patterns of children and socioeconomical, behavioral and maternal determinants. Rev. Paul. Pediatr..

[66] Van Ansem W.J., Schrijvers C.T., Rodenburg G., Van de Mheen D. (2014). Maternal educational level and children’s healthy eating behaviour: Role of the home food environment (cross-sectional results from the INPACT study). Int. J. Behav. Nutr. Phys. Act..

[67] Parks E.J., Skokan L.E., Timlin M.T., Dingfelder C.S. (2008). Dietary sugars stimulate fatty acid synthesis in adults. J. Nutr..

[68] Kennedy A., Martinez K., Chuang C.C., LaPoint K., McIntosh M. (2009). Saturated fatty acid-mediated inflammation and insulin resistance in adipose tissue: Mechanisms of action and implications. J. Nutr..

[69] Malik V.S., Pan A., Willett W.C., Hu F.B. (2013). Sugar-sweetened beverages and weight gain in children and adults: A systematic review and meta-analysis. Am. J. Clin. Nutr..

[70] Scholz A., Navarrete-Muñoz E.M., García-de-la-Hera M., Fernandez-Somoano A., Tardon A., Santa-Marina L., Pereda-Pereda E., Romaguera D., Guxens M., Beneito A. (2019). Association between trans fatty acid intake and overweight including obesity in 4 to 5-year-old children from the INMA study. Pediatr. Obes..

[71] Hu F.B. (2002). Dietary pattern analysis: A new direction in nutritional epidemiology. Curr. Opin. Lipidol..

[72] Brakenhoff T.B., Van Smeden M., Visseren F.L.J., Groenwold R.H.H. (2018). Random measurement error: Why worry? An example of cardiovascular risk factors. PLoS ONE.

[73] Keogh R.H., Shaw P.A., Gustafson P., Carroll R.J., Deffner V., Dodd K.W., Küchenhoff H., Tooze J.A., Wallace M.P., Kipnis V. (2020). STRATOS guidance document on measurement error and misclassification of variables in observational epidemiology: Part 1—Basic theory and simple methods of adjustment. Stat. Med..

[74] Talbot D., Diop A., Lavigne-Robichaud M., Brisson C. (2021). The change in estimate method for selecting confounders: A simulation study. Stat. Methods Med. Res..

