# Supplementary material for: Consumption and Breakfast Patterns in Children and Adolescents with Congenital Heart Disease
Source: Int J Environ Res Public Health. 2023 Mar 15;20(6):5146. doi: 10.3390/ijerph20065146 (PMC10048830; doi:10.3390/ijerph20065146)
Supplement: Supplementary file 1 [file ijerph-20-05146-s001.zip › ijerph-2090045-supplementary.pdf]

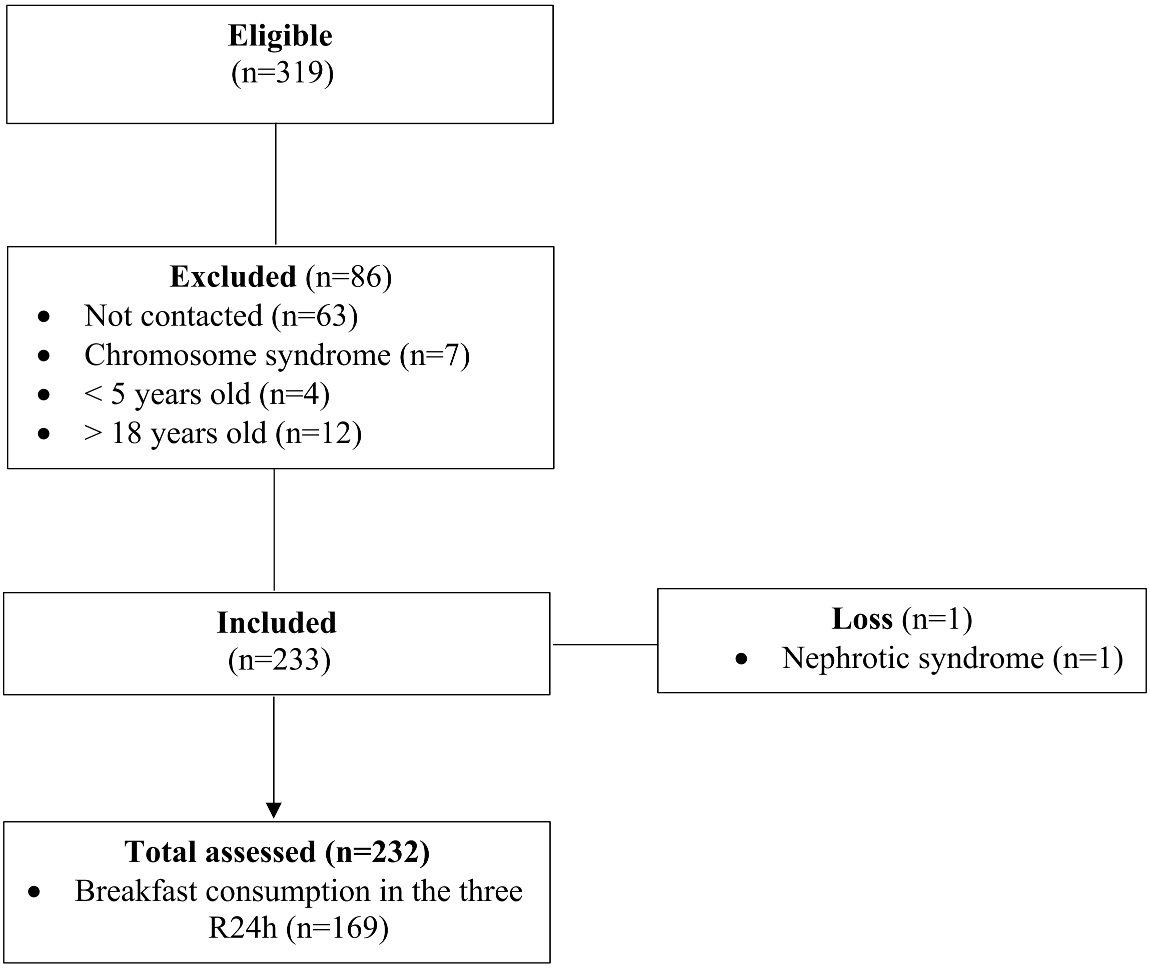

Figure S1. Flowchart of the selection of children and adolescents with CHD. Florianópolis, Southern Brazil, 2017.
